# Interspinous process devices for treatment of degenerative lumbar spine stenosis: A systematic review and meta-analysis

**DOI:** 10.1371/journal.pone.0199623

**Published:** 2018-07-06

**Authors:** Arthur Werner Poetscher, Andre Felix Gentil, Mario Ferretti, Mario Lenza

**Affiliations:** Hospital Israelita Albert Einstein, São Paulo, SP, Brazil; Dartmouth-Hitchcock Medical Center, UNITED STATES

## Abstract

**Background:**

Degenerative lumbar spinal stenosis is a condition related to aging in which structural changes cause narrowing of the central canal and intervertebral foramen. It is currently the leading cause for spinal surgery in patients over 65 years. Interspinous process devices (IPDs) were introduced as a less invasive surgical alternative, but questions regarding safety, efficacy, and cost-effectiveness are still unanswered.

**Objectives:**

The aim of this study was to provide complete and reliable information regarding benefits and harms of IPDs when compared to conservative treatment or decompression surgery and suggest directions for forthcoming RCTs.

**Methods:**

We searched MEDLINE, EMBASE, Cochrane Library, Scopus, and LILACS for randomized and quasi-randomized trials, without language or period restrictions, comparing IPDs to conservative treatment or decompressive surgery in adults with symptomatic degenerative lumbar spine stenosis. Data extraction and analysis were conducted following the Cochrane Handbook. Primary outcomes were pain assessment, functional impairment, Zurich Claudication Questionnaire, and reoperation rates. Secondary outcomes were quality of life, complications, and cost-effectiveness. This systematic review was registered at Prospero (International prospective register of systematic reviews) under number 42015023604.

**Results:**

The search strategy resulted in 17 potentially eligible reports. At the end, nine reports were included and eight were excluded. Overall quality of evidence was low. One trial compared IPDs to conservative treatment: IPDs presented better pain, functional status, quality of life outcomes, and higher complication risk. Five trials compared IPDs to decompressive surgery: pain, functional status, and quality of life had similar outcomes. IPD implant presented a significantly higher risk of reoperation. We found low-quality evidence that IPDs resulted in similar outcomes when compared to standard decompression surgery. Primary and secondary outcomes were not measured in all studies and were often published in incomplete form. Subgroup analysis was not feasible. Difficulty in contacting authors may have prevented us of including data in quantitative analysis.

**Conclusions:**

Patients submitted to IPD implants had significantly higher rates of reoperation, with lower cost-effectiveness. Future trials should improve in design quality and data reporting, with longer follow-up periods.

## Introduction

Degenerative lumbar spinal stenosis (DLSS) is a condition related to aging in which changes in facet joints, ligamentum flavum, posterior longitudinal ligament and intervertebral disc cause narrowing of the central canal and intervertebral foramen [1].

Central canal narrowing is mostly associated to neurogenic claudication, while lateral recess and intervertebral foramen stenosis usually present with radicular syndromes. Back pain is usually present. Degenerative listhesis may also be associated, with or without instability. These clinical features may be considered separately or together, creating heterogeneous cohorts[2, 3].

Despite the absence of clear diagnostic and classification criteria, symptomatic DLSS is relatively common. Kalichman et al. [4], using data from the Framington study, found a prevalence of radiologic DLSS ranging from 19% to 47%, depending on criteria used. Indeed, DLSS is currently the leading cause for spinal surgery in patients over 65 years[5].

When nonsurgical treatment fails (e.g. physical therapy, spine injections, conventional and neuropathic pain medicine) and the patient is in pain and functionally limited, surgical treatment may be an option. Decompression seems to be particularly beneficial when radicular pain and/or neurogenic claudication are the predominant symptoms [2]. The classic surgical approach consists of a wide decompression, associated or not to arthrodesis. Morbidity includes post-operative pain, dural tear, blood loss, infection, and immobilization [3].

Cadaveric spine studies suggested that an interspinous process device (IPD) could improve the central canal area in up to 18% [6]. At the beginning of this century, IPDs were approved for patient use [6] and introduced as a less invasive surgical alternative. Questions regarding safety, efficacy, and cost-effectiveness are still unanswered. Randomized controlled trials (RCT) have compared IPDs to conservative treatment [7–9] or to standard surgery[10–16], but there is an overall concern regarding bias risks and small population samples. Four systematic reviews have been published[17–20], but only one of these reviews examined exclusively RCTs[19]. Nevertheless, this systematic review included only 4 of the available studies.

The aim of this study was to provide complete and reliable information regarding benefits and harms of IPDs when compared to conservative treatment or decompression surgery and suggest directions for forthcoming RCTs.

## Methods

This systematic review was registered at the institution's research management system under number 2287–15 and at Prospero (International prospective register of systematic reviews) under number 42015023604. We followed the PRISMA statement for systematic reviews guidelines[21]. The PRISMA checklist may be found in S1 Appendix.

### Patients, interventions, comparators and outcomes

#### Patients

Male and female adults with DLSS confirmed by computed tomography scan (CT) or MRI were included. Patients with revision surgeries or previous surgeries were excluded.

#### Intervention

Treatment of DLSS by the means of any brand / model of IPD.

#### Comparators

Nonsurgical treatment or conventional surgery (decompression with or without arthrodesis). Studies that compared IPDs without a control condition were excluded.

#### Primary outcomes

1. pain (visual analog scale [VAS], numeric scales); 2. functional impairment (Oswestry Disability Inventory [ODI], Roland-Morris Questionnaire [RMQ]); 3. Specific DLSS scales (Zurich Claudication Questionnaire [ZCQ]) [22]; 4. reoperation rates.

#### Secondary outcomes

5. quality of life (SF-36, EuroQol); 6. complications (neurologic injuries, bleeding, fractures, infection); 7. Cost-effectiveness.

### Search strategy

We searched MEDLINE /PubMed (November, 1990 to August 3^rd^, 2017), EMBASE (February, 1995 to August 3^rd^, 2017), Cochrane Library (March, 1987 to August 3^rd^, 2017), Scopus (December 2003 to August 3^rd^, 2017), and LILACS (April, 1987 to August 3^rd^, 2017) for randomized and quasi-randomized trials, without language or period restrictions. This was the search strategy for MEDLINE (PubMed): (Spinal Stenosis [mh] OR Low Back Pain [mh] OR spondylosis, lumbarsacral [mh] OR neurologic claudication* [tw]) AND (Prostheses and Implants [mh] OR interspinous device*[tw] OR interspinous spacer*[tw]) AND (randomized controlled trial [pt] OR controlled clinical trial [pt] OR randomized controlled trials [mh] OR random allocation [mh] OR double-blind method [mh] OR single-blind method [mh] OR clinical trial [pt] OR clinical trials [mh] OR (“clinical trial” [tw]) OR ((singl* [tw] OR doubl* [tw] OR trebl* [tw] OR tripl* [tw]) AND (mask* [tw] OR blind* [tw])) OR (placebos [mh] OR placebo* [tw] OR random* [tw] OR research design [mh:noexp]) NOT (animals [mh] NOT humans [mh])).

### Data extraction

Two authors (AWP e AFG) extracted data identifying study and participant characteristics, types of interventions, outcome measures, and methodology. Input from a third author (ML) was used when deemed necessary. We attempted to obtain missing information by directly contacting authors of primary studies (i.e. number of participants, dropout details, means, measures of uncertainty, or number of events).

### Bias assessment

Following the registered protocol, risk of bias assessment was independently conducted by two authors (AWP e AFG). The following domains were considered: 1. random sequence generation; 2. allocation concealment; 3. blinding of participants and personnel; 4. blinding of outcome assessment; 5. incomplete outcome data; 6. selective reporting; 7. other bias (e.g. unbalanced demographic data, inappropriate funding, etc.). Each item was classified for the risk of bias as low, high, or unclear[23].

### Data analysis

Risk ratios (RR) were calculated with 95% confidence intervals (CI) for dichotomous outcomes. Treatment effects for continuous outcome data were expressed as mean differences (MD) with 95% CI. Sensitivity analysis was performed to examine the effects of pooled data. Heterogeneity of estimate effects between included studies was assessed by visual inspection of the forest plot and the test for heterogeneity. The magnitude of inconsistency was quantified across studies using the I-squared statistic, interpreted as follows: 0% to 40% might not be important; 30% to 60% may represent moderate heterogeneity; 50% to 90% may represent substantial heterogeneity; 75% to 100% considerable heterogeneity. In cases of considerable heterogeneity, we explored the data further by comparing the characteristics of individual studies and conducted subgroup analyses. When appropriate, results of comparable groups of studies were pooled in meta-analysis using the random-effect model as a default (considering cohort heterogeneity). For dichotomous outcomes we calculated pooled RR with 95% CI. When two or more studies presented continuous data derived from the same validated instrument of evaluation using the same units of measure, data were pooled as MD with 95% CI. When primary studies expressed the same variable using different instruments and different units of measure, we used the standardized mean difference (SMD) with 95% CI. When necessary, standard deviation (SD) was calculated from: 1. standard error (SE); 2. CI and sample size; or 3—*p* value for a t-test comparing 2 means.

1. SD=SE√N
2. SD=Nx(upperlimit−lowerlimit)3.92
3. Given *p* value and both sample sizes, we recovered the bicaudal inverse of Student t-function, based on N1+N2–2 levels of freedom (INV T BC function on Excel).

Then: SE=MDt and SD=SE1N1+1N2, assuming that the mean of SD’s may represent both values(23).

The compiled dataset was analyzed with a statistical software package (Review Manager® Version 5.3. Copenhagen: The Nordic Cochrane Centre, the Cochrane Collaboration, 2012).

We used the GRADE approach to assess the quality of evidence related to each of the key outcomes[24]. We reported the main results of our review in a 'Summary of findings' table. The 'Summary of findings' table makes available key information concerning the quality of evidence, the magnitude of effect of the interventions examined, and the sum of available data on the main outcomes.

## Results

### Search strategy

**Fig 1:**
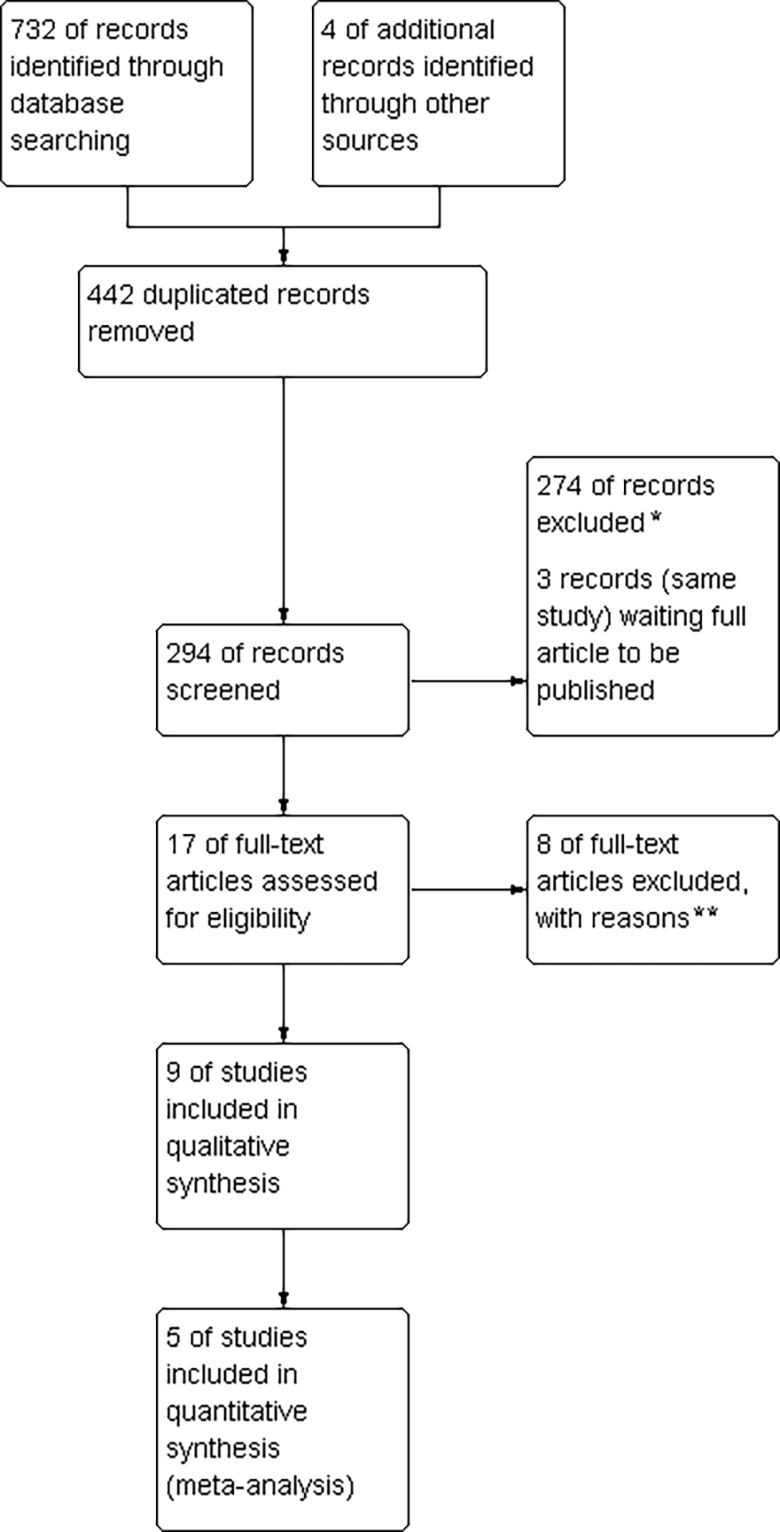
Study flow diagram: Of 732 identified records, 9 studies qualified for qualitative analysis and 5 for meta-analysis. *reason for exclusion: did not meet all inclusion criteria; ** reason for exclusion listed in Table 1.

Our search strategy identified a total of 736 records. After removing repeated inputs, 294 records were reviewed, and full articles were obtained for 17 potentially eligible reports published between 2005 and 2015. At the end of the assessment, six studies were included (nine reports) and eight were excluded (Fig 1).

### Patients and study characteristics

We rejected eight papers[25–32] for several reasons, most frequently for not being randomized or for the absence of a control group (Table 1).

**Table 1:** Description of excluded studies.

Study	Reason for exclusion
Beyer 2013 [25]	Not randomized
Kong 2007 [26]	Retrospective, not randomized, case-control study
Lauryssen 2015 [27]	Compared 2 devices with historical series
Marsh 2014 [28]	Compared IPD + decompression to decompression
Patel 2015 [29]	No control group, compared 2 devices
Puzzilli 2014 [30]	Included patients with black disc disease and facet degeneration without stenosis
Skidmore 2011 [31]	Compared IPD to decompression in a subgroup of patients that failed control treatment
Sobotke 2010 [32]	Not randomized

IPD = interspinous process device

The six studies included were RTCs, with five being multicentric. The minimum age for recruitment was 20 years in one trial[10]; 40 years in three trials[11, 14, 15] and being older than 50 in two other trials[9, 13].

One study compared the IPD to non-surgical treatment[7–9] and therefore excluded patients with more severe symptoms for the control condition to be ethically acceptable. Among the studies that used standard surgery as control group, one study[11] excluded patients with mild symptoms. Details of included studies can be found in Table 2.

**Table 2:** Description of included studies.

Study	Study Design	Population	Population (exclusion)	Intervention / control	Outcomes
Anderson/ Hsu/ Zucherman 2005 [7–9]	RCT, multicenter	-Mean age 70 (IPD), 69.1 (control) -1.2 female/ male (IPD), 2 female / male (control) -Clinical or radiographic DLSS confirmation - 1 or 2 levels affected -Able to sit 50 min and walk > 50 ft (15.24m) - Nonoperative treatment > 6 mo	-Fixed motor deficit / cauda equine syndrome -Previous lumbar surgery -Significant instability or scoliosis -Pathological fractures or severe osteoporosis -Obesity -Active infection or systemic disease -Paget's disease or metastasis to the vertebrae -Steroid use > 1 month during last 12 months	IPD (X-Stop) vs. non-surgical treatment	-ZCQ -SF-36 -Patient satisfaction
Azzazzi 2010 [10]	RCT, single center	-Mean age 57 (IPD), 56.3 (control) -2.75 female / male (IPD), 1.73 female / male (control) -DLSS + grade I listhesis -1 or 2 affected levels -At least moderate disability -Leg pain > back pain -Nonoperative treatment > 3 mo	-Previous lumbar surgery - Osteopenia / osteoporosis - Obesity (BMI > 40) -Active infection, systemic disease or malignancy	IPD (X-Stop) vs. surgery (decompression and arthrodesis)	-VAS back pain -VAS leg pain -ODI
Davis 2013 [11]	RCT, multicenter	-Mean age 62.1 (IPD), 64.1 (control) -Neurogenic claudication and radiographic confirmation of DLSS -1 or 2 affected levels -VAS back pain > 50 -ODI > 20/50	-Fixed motor deficit -Previous lumbar surgery -Significant instability or scoliosis -Pathological fractures or osteopenia -Paget's disease -Obesity (BMI > 40) -Active infection, systemic disease or malignancy -Axial back pain only -Significant peripheral neuropathy or vascular disease - Need for discectomy -Seeking or receiving worker's compensation -Substance abuse	IPD (coflex) vs. surgery (decompression and arthrodesis)	-ZCQ -VAS back pain -VAS leg pain -ODI -SF-12
Lonne 2015 [12, 13]	RCT, multicenter	-Mean age 67 (IPD), 67 (control) - 1.35 female / male (IPD), 0.75 (control) -Neurogenic claudication and DLSS confirmation on MRI -1 or 2 affected levels -up to grade I listhesis	-Severe motor deficit / cauda equine syndrome -Previous lumbar surgery -Significant instability or scoliosis -Osteoporosis -Significant peripheral neuropathy or vascular disease -Unilateral radiculopathy -ASA > 3 or malignancy	IPD (X-Stop) vs. surgery (minimally invasive decompression)	-ZCQ -Numerical pain scale -ODI -EQ-5D -QALY
Moojen / Marle 2015 [14, 16]	RCT, multicenter	-Median age 66 (IPD), 64 (control) -0.63 female / male (IPD), 1.16 female / male (control) -neurogenic claudication and DLSS confirmation on MRI -1 or 2 affected levels	-Cauda equine syndrome -Previous lumbar surgery -Significant instability or scoliosis -Need for discectomy	IPD (coflex) vs. surgery (decompression)	-ZCQ -VAS back pain -VAS leg pain -McGill pain questionnaire -RMQ -SF-36 -HADS -Shuttle walking test
Strömkvist 2013 [15]	RCT, multicenter	-mean age 67 (IPD), 71 (control) -0.67 female / male (IPD), 0.92 female / male (control) -neurogenic claudication and DLSS confirmation on MRI -> 6 mo symptoms -1 or 2 affected levels -up to grade 1 listhesis	-Previous lumbar surgery -L5-S1 level -Infection or malignancy -Osteoporosis	IPD (X-Stop) vs. surgery (decompression)	-ZCQ -VAS back pain -VAS leg pain -SF-36

BMI = body mass index, MRI = magnetic resonance image, DLSS = degenerative lumbar spine stenosis, EQ-5D = EuroQol five dimension scale, IPD = interspinous process device, ODI = Oswestry Disability Inventory, RCT = randomized controlled trial, SF-12 = Medical Outcomes Study 12-Item Short-Form Health Survey, SF-36 = Medical Outcomes Study 36-Item Short-Form Health Survey, VAS = visual analogue scale, ZCQ = Zurich Claudication Questionnaire

### Bias assessment

**Fig 2:**
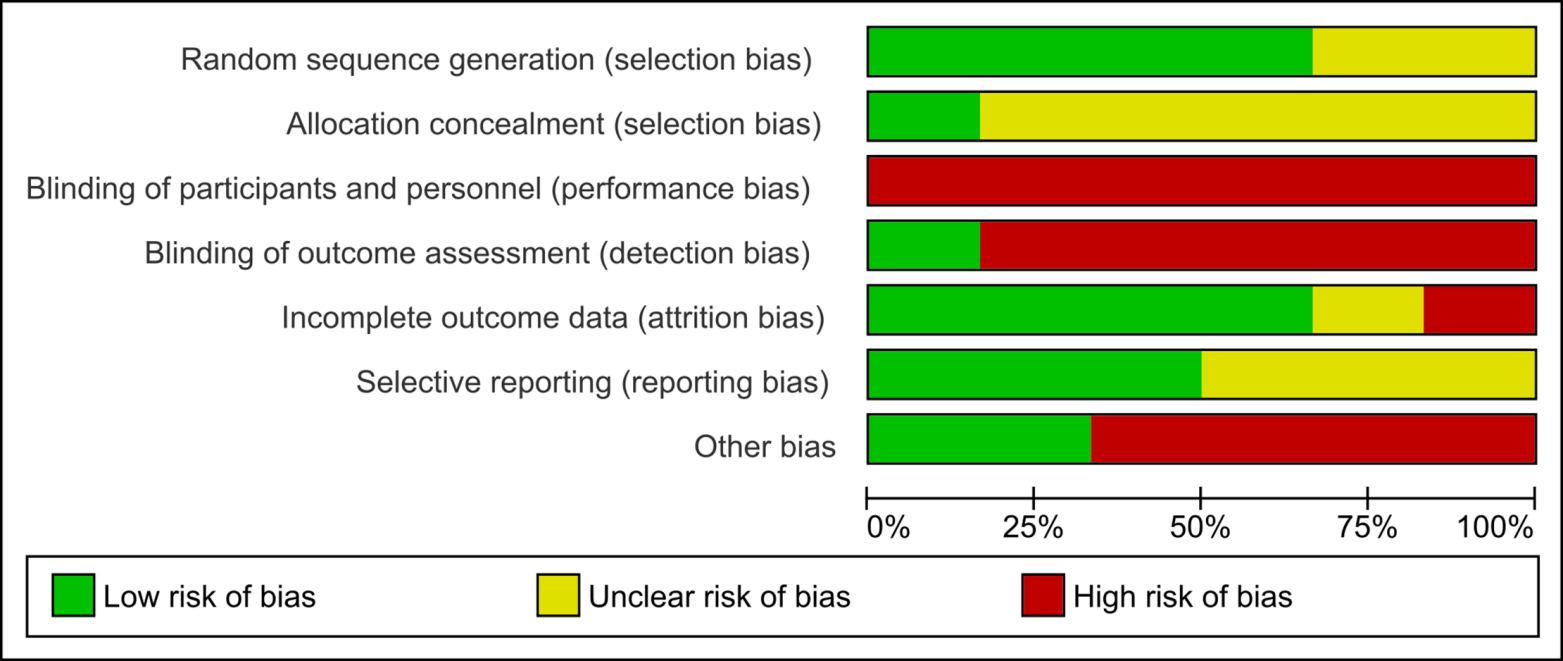
Risk of bias graph: Overall risk of bias for included studies, demonstrating high performance and detection bias for almost all trials according to the review authors' judgments.

**Fig 3:**
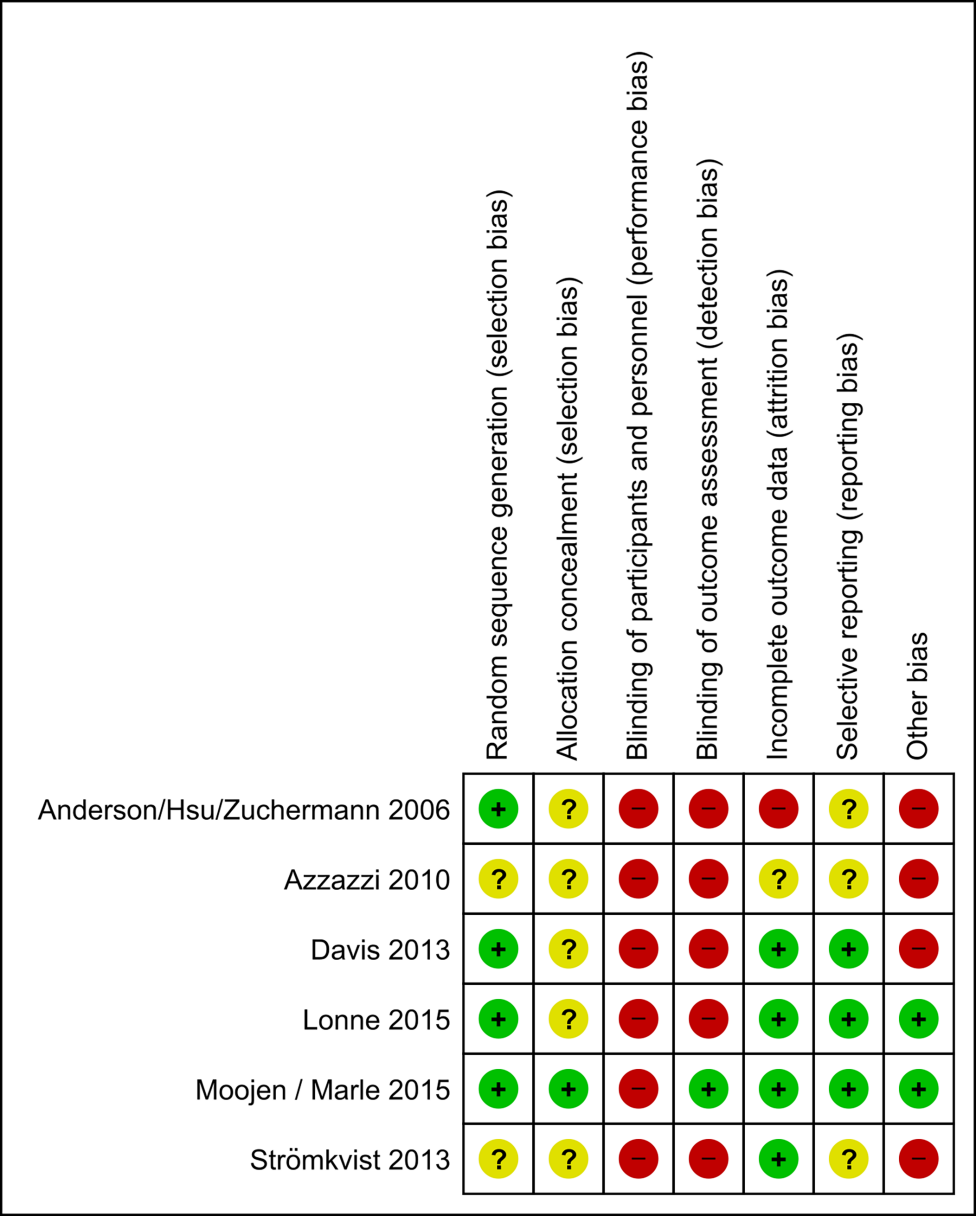
Risk of bias summary: Authors' judgements review about each risk of bias item for each included study.

Methodological flaws were the rule and not the exception in many trials, and assessment revealed an overall high risk of bias, especially concerning blinding of participants and personnel (performance bias) and blinding of outcome assessment (detection bias). The authors’ judgments about bias risks for each study are presented in Figs 2 and 3.

### Effects of interventions

The primary aim of this review was to investigate the possible benefits and harms of IPDs for the treatment of DLSS compared to nonsurgical treatments or decompressive surgery. The studies included allowed the analysis of both comparisons: IPD versus conservative treatment [7–9]and IPD versus decompressive surgery[10–16].

#### IPD vs. conservative treatment

The reports of Zucherman et al[9], Anderson et al[7] and Hsu et al[8] refer to the same trial. Non-surgical treatment consisted in spinal injections, anti-inflammatory and analgesic drugs, and physical therapy. IPD was implanted with standard procedure.

#### Primary outcomes

Pain and overall functional status were not assessed in any report.

The specific ZCQ was included in two papers. Zucherman et al[9] and Anderson et al[7] (reporting on a subgroup of patients with listhesis) concluded that patients in the IPD group improved significantly more than controls in all three ZCQ domains.

Treatment failure was defined as need for laminectomy in both groups (reoperation in the IPD group). A total of six patients out of 93 in the IPD group needed laminectomy, compared to 24 out of 81 in the control condition (RR 0.22 [0.09, 0.51]). Considering scenarios where all missing patients failed treatment (RR 0.57 [0.33, 0.96]) or no one failed (RR 0.25 [0.11, 0.59]), treatment failure was consistently higher with conservative treatment. The subgroup of patients with listhesis published by Anderson et al[7] had similar outcomes.

#### Secondary outcomes

Hsu et al[8] analyzed quality of life (SF-36) changes in patients submitted to IPD implants compared to controls. He reported a significant improvement in the IPD group, especially in domains related to physical activity.

Complications were most frequent in the IPD group (11 in 93 patients vs. 6 in 81 for controls, RR 1.60 [0.62, 4.12]). Considering scenarios where all missing patients presented with complications (RR 1.02 [0.56, 1.89]) or no one did (RR 1.67 [0.64, 4.33]), complications were more frequent in the IPD group.

Again, the subgroup of patients with listhesis published by Anderson et al[7] had similar outcomes.

The trial reported by Zucherman et al(9[9]), Anderson et[7] al and Hsu et al[8] did not provide analysis on cost-effectiveness.

#### IPD vs. decompressive surgery: Primary outcomes

**Fig 4:**
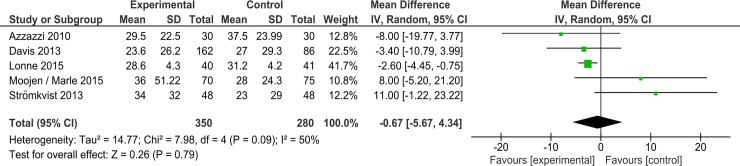
Comparison II (device versus decompressive surgery interventions), outcome VAS back pain. Meta-analysis revealed no significant difference between groups.

Azzazzi et al[10] observed that at the end of follow up (24 months) the IPD group back pain VAS improved to a mean of 29.5 points, while the control group improved to a mean of 37.5. Davis et al[11] found at 24 months a mean back pain of 23.6 for IPD and 27 for control patients (p = 0.345, effect size -0.13). Lonne et al[13] reported at 2 years mean back pain scores of 28.6 for the IPD group and 31.2 for the control group (p = 0.658). Moojen et al[14] observed at 104 weeks back pain VAS scores of 36 in the IPD group and 28 in the control group (p = 0.26). Stromkvist et al[15] found at 24 months mean back pain VAS of 34 for IPD and 23 for control patients. Results of meta-analysis are presented in Fig 4.

**Fig 5:**
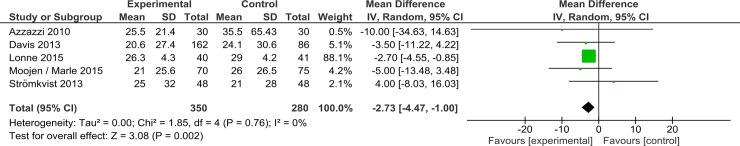
Comparison II (device versus decompressive surgery interventions), outcome VAS leg pain. Pooled data favors IPD, but the difference was not clinically significant.

Azzazzi et al[10] observed that at the end of follow up (24 months) the IPD group leg pain improved to a mean fo 25.5 points, while the control group improved to a mean of 35.5. Daviset al[11] found at 24 months a mean leg pain of 20.6 for IPD and 24.1 for control patients (p = 0.364, effect size -0.12). Lonne et al[13] reported at 2 years mean leg pain scores of 26.3 for the IPD group and 29 for control patients (p = 0.65). Moojen et al[14] observed at 104 weeks leg pain scores of 21 in the IPD group and 26 in the control group (p = 0.22). Stromkvist et al[15] found at 24 months a mean leg pain VAS of 25 for IPD and 21 for control patients. Quantitative synthesis is presented in Fig 5.

**Fig 6:**
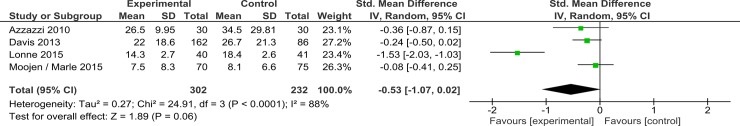
Comparison II (device versus decompressive surgery interventions), outcome overall functional status. Meta-analysis revealed no significant difference between groups and high heterogeneity.

**Fig 7:**
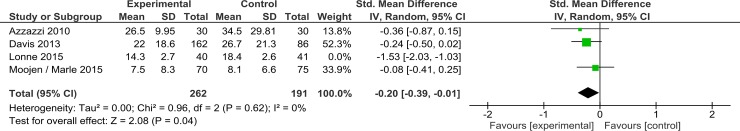
Comparison II (device versus decompressive surgery interventions), sensitivity analysis for the outcome of overall functional status without data from Lonne, showing high homogeneity.

Azzazzi et al[10] observed that at the end of follow up (24 months) the IPD group ODI improved to 26.5 points (mean), while control group improved to 34.5 (mean). Davis et al[11] found at 24 months a mean ODI of 22 for IPD and 26.7 for control patients (p = 0.075, effect size -0.24). Lonne et al[13] reported at 2 years mean ODI scores of 14.3 for the IPD group and 18.4 for the control group (p = 0.285). Moojen et al[14] observed for Modified Roland Disability Questionnaire at 24 months a score of 7.5 in the IPD group and 8.1 in the control group (p = 0.65). Stromkvist et al[15] did not use non-specific functional scales. Meta-analysis for overall functional status (Fig 6) resulted in a SMD of -0.53 [-1.07, 0.02] with high heterogeneity (I^2^ = 88%). In sensitivity analysis (Fig 7), we excluded data from Lonne et al[13] (the only study where the control group used a minimally invasive surgical technique) and SMD scores changed to -0.20 [-0.39, -0.01], I^2^ = 0%.

Davis et al[11] reported the following mean scores for ZCQ at 24 months of follow-up: symptom severity 1.98 (IPD) vs. 0.89 (control) (p = 0.023, effect size -0.30); physical function 1.56 (IPD) vs. 0.77 (control) (p = 0.008, effect size -0.35); patient satisfaction 0.55 (IPD) vs. 0.77 (control) (p = 0.006, effect size -0.036). Lonne et al[13] observed at 2 years the following mean results: symptom severity 2.20 (IPD) vs. 2.42 (control) (p = 0.234); physical function 1.65 (IPD) vs. 1.66 (control) (p = 0.922); patient satisfaction 1.75 (IPD) vs. 1.89 (control) (p = 0.477). Moojen et al[14] reported an overall ZCQ successful improvement in 69 out of 70 patients for IPD and 60 out of 75 in the control group (OR 0.65, p = 0.20). Stromkvist et al[15] observed no difference between groups in ZCQ scores after 24 months. Meta-analysis with data from Davis et al[11] and Lonne et al[13] revealed a mean difference of -0.22 [CI 95% -0.28, -0.17] for symptom severity, -0.02 [-0.06, 0.02] for physical function, and -0.15 [-0.20, -0.09] for patient satisfaction.

**Fig 8:**
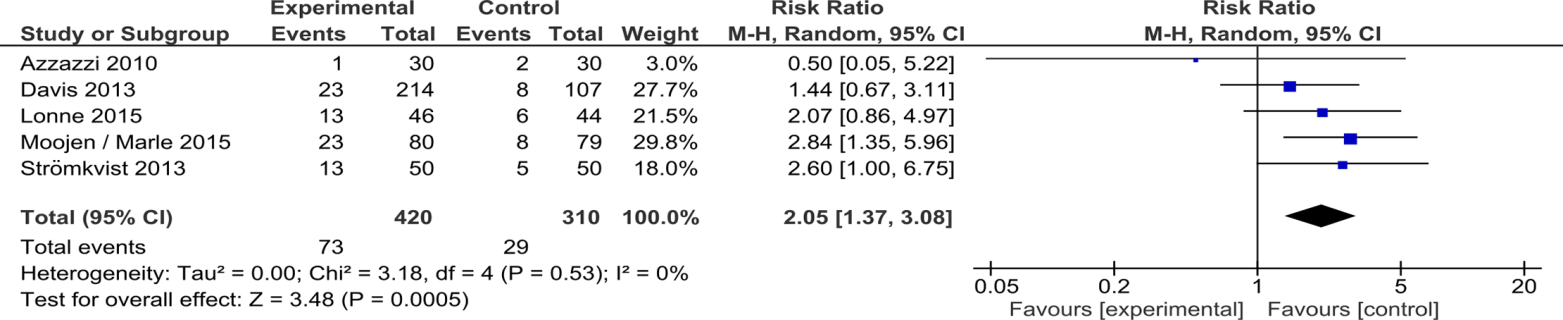
Comparison II device versus decompressive surgery interventions, outcome treatment failure (intention to treat). Meta-analysis revealed significant high risk ratio of reoperation in IPD group.

The overall reoperation rate was higher in the IPD group in all studies, with the trial from Azzazzi et al[10] being the only exception, reporting a risk ratio of 0.50 [0.05, 5.22]. Among the other studies, risk ratios ranged from 1.53 [0.71, 3.27] [4] to 3.08 [1.48, 6.43] [19]. Lonne et al[12, 13] interrupted recruiting after midterm evaluation due to high rates of reoperation secondary to persistent or recurrent symptoms in the IPD group. Meta-analysis for these findings is detailed in Fig 8. Considering scenarios where all missing patient failed treatment (RR 1.79 [1.23, 2.61]) or no one failed (RR 2.05 [1.37, 3.08]), treatment failure was consistently higher in the IPD group.

#### Secondary outcomes

Davis et al[11] measured the physical and mental health components of SF-12 and found for the physical component at 24 months a mean score of 43.8 for patients in the IPD group and 40.7 for controls (p = 0.05, effect size 0.28). There was no significant difference for health component. Lonne et al[13] applied EQ-5D and reported at 2 years mean scores of 0.688 for the IPD group and 0.73 for the control group (p = 0.416). Moojen et al[14] used SF-36 and reported for physical functioning a mean score of 63 for IPD patients and 62 for controls after 54 weeks of follow-up, with results being similar in other domains. Stromqvist et al[15] measured the SF-36 physical component score and found no difference between groups at 24 months. Pooled data from 4 studies resulted in a standard mean difference of 0.32 [not significant, -0.02, 0.65], with high heterogeneity (I^2^ = 71%). Sensitivity analysis did not reduce heterogeneity.

Four studies reported complications other than reoperation. Azzazzi et al[10], Moojen et al[14] and Stromkvist et al[15] considered only direct surgical complications, whereas Davis et al[11] used more ample criteria. Risk ratio ranged from 0.17 [0.04, 0.68] [2] to 1.34 [0.37, 4.79] [19]. Meta-analysis resulted in a risk ratio of 0.66 [0.24, 1.83], with high heterogeneity (I^2^ = 68%). Sensitivity analysis reduced heterogeneity significantly when removing Azzazzi’s et al[10] study, which was classified with higher bias risk (RR 1.19 [0.95, 1.48], I^2^ = 0%). Considering scenarios where all missing patient failed treatment (RR 0.86 [0.41, 1.81]) or no one failed (RR 0.65 [0.25, 1.69]), differences were not significant.

Outcomes of Davis et al[11] were later published in shorter form, and with 5 years of follow-up results were very similar([33].

Van den Akker-van Marle et al[16] analyzed the same data set as Moojen et al (FELIX Trial)[14] regarding QALY, health-care costs, social costs, and a cost-utility analysis. They observed that the probability of IPDs being more cost effective than decompression was far below 50%. The costs of IPDs were significantly higher and did not improve quality of life when compared to surgical decompression.

Lonne et al[12] published cost-effectiveness data in a separate paper, comparing health outcome, health care costs and calculating the incremental cost-effectiveness ratio: (total cost IPD–total cost control) / (QALY IPD–QALY control). The conclusion was that IPD was 50% more likely to be cost effective only at an extra-cost of Euro 25,700. The higher cost of IPDs were a consequence of implant cost and higher reoperation rate.

## Discussion

### Summary of main results

We included six trials that resulted in nine papers involving 930 patients to analyze effectiveness and safety of IPD implant for treating DLSS. One trial compared IPD to conservative treatment, and five trials compared IPD to decompressive surgery.

Overall trial quality was low, and there was missing data for important outcomes from all trials.

One trial suggested that IPD is more effective than conservative treatment for DLSS[7–9]. Treatment failure appeared to be significantly lower in the implant group. However, complications seemed to be more frequent for the IPD group compared to conservative treatment.

Low quality evidence indicated that outcomes regarding pain, functional status and quality of life are similar. However, treatment failure was significantly higher in IPD group compared to decompressive surgery. Results are conflicting for other complications. Cost-effectiveness analysis favored conventional surgery. Summary of results according to GRADE criteria can be found in Tables 3 and 4.

**Table 3:** Certainty assessment of evidence.

Certainty assessment							№ of patients		Effect		Certainty	Importance
№ of studies	Study design	Risk of bias	Inconsistency	Indirectness	Imprecision	Other considerations	Device	Surgery	Relative (95% CI)	Absolute (95% CI)		
Zurich Claudication Questionnaire–Symptom Severity												
2	randomized trials	serious	not serious	not serious	serious	none	201	127	-	MD **0.22 lower** (0.28 lower to 0.17 lower)	⨁⨁◯◯ LOW	CRITICAL
Zurich Claudication Questionnaire–Physica Function												
2	randomized trials	serious	very serious	not serious	serious	none	202	127	-	MD **0.02 lower** (0.06 lower to 0.02 higher)	⨁◯◯◯ VERY LOW	CRITICAL
Zurich Claudication Questionnaire Satisfaction												
2	randomized trials	serious	not serious	not serious	serious	none	202	127	-	MD **0.15 lower** (0.2 lower to 0.09 lower)	⨁⨁◯◯ LOW	CRITICAL
Visual Analog Scale–back pain												
5	randomized trials	serious	serious	not serious	not serious	none	350	280	-	MD **0.67 lower** (5.67 lower to 4.34 higher)	⨁⨁◯◯ LOW	CRITICAL
Visual Analog Scale—leg pain												
5	randomized trials	serious	not serious	not serious	serious	none	350	280	-	MD **2.73 lower** (4.47 lower to 1 lower)	⨁⨁◯◯ LOW	CRITICAL
Overall Functional Status												
4	randomized trials	serious	serious	not serious	not serious	none	302	232	-	SMD **0.53 lower** (1.07 lower to 0.02 higher)	⨁⨁◯◯ LOW	CRITICAL
Quality of Life												
4	randomized trials	serious	serious	not serious	not serious	none	299	235	-	SMD **0.32 higher** (0.02 lower to 0.65 higher)	⨁⨁◯◯ LOW	IMPORTANT
Complications (patients treated)												
3	randomized trials	serious	not serious	not serious	not serious	none	112/280 (40.0%)	54/209 (25.8%)	**RR 1.19** (0.95 to 1.48)	**49 more per 1.000** (from 13 fewer to 124 more)	⨁⨁⨁◯ MODERATE	IMPORTANT
Complications (patients allocated)												
3	randomized trials	serious	not serious	not serious	not serious	none	112/344 (32.6%)	54/236 (22.9%)	**RR 1.11** (0.87 to 1.43)	**25 more per 1.000** (from 30 fewer to 98 more)	⨁⨁⨁◯ MODERATE	IMPORTANT
Reoperations (patients treated)												
5	randomized trials	serious	not serious	not serious	serious	none	73/350 (20.9%)	29/280 (10.4%)	**RR 2.17** (1.45 to 3.24)	**121 more per 1.000** (from 47 more to 232 more)	⨁⨁◯◯ LOW	CRITICAL
Reoperations (patients allocated)												
5	randomized trials	serious	not serious	not serious	serious	none	73/420 (17.4%)	29/310 (9.4%)	**RR 2.05** (1.37 to 3.08)	**98 more per 1.000** (from 35 more to 195 more)	⨁⨁◯◯ LOW	CRITICAL

**Table 4:** Summary of findings.

Outcomes	Anticipated absolute effects* (95% CI)		Relative effect (95% CI)	№ of participants (studies)	Certainty of the evidence (GRADE)	Comments
	Risk with Surgery	Risk with Device				
ZCQ-SS	-	The mean ZCQ-S in the intervention group was 0,22 lower (0,28 lower to 0,17 lower)	-	328 (2 RCTs)	⨁⨁◯◯ LOW	Not clinically significant
ZCQ-PF	-	The mean ZCQ-PF in the intervention group was 0,02 lower (0,06 lower to 0,02 higher)	-	329 (2 RCTs)	⨁◯◯◯ VERY LOW	Not clinically significant
ZCQ Satisfaction	-	The mean ZCQ Satisfaction in the intervention group was 0,15 lower (0,2 lower to 0,09 lower)	-	329 (2 RCTs)	⨁⨁◯◯ LOW	Not clinically significant
VAS–back pain	-	The mean VAS—backpain in the intervention group was 0,67 lower (5,67 lower to 4,34 higher)	-	630 (5 RCTs)	⨁⨁◯◯ LOW	Not clinically significant
VAS—leg pain	-	The mean VAS—leg in the intervention group was 2,73 lower (4,47 lower to 1 lower)	-	630 (5 RCTs)	⨁⨁◯◯ LOW	Not clinically significant
Overall Functional Status	-	The standard mean difference for overall functional status was -0.53 [-1.07 to- 0.02] favoring IPD group	-	534 (4 RCTs)	⨁⨁◯◯ LOW	Not clinically signifficant
Quality of Life	-	The standard mean difference for overall quality of life was 0.32 [-0.02 to 0.65] favoring IPD group	-	534 (4 RCTs)	⨁⨁◯◯ LOW	Not clinically signifficant
Complications (patients treated)	258 per 1.000	307 per 1.000 (245 to 382)	RR 1.19 (0.95 to 1.48)	489 (3 RCTs)	⨁⨁⨁◯ MODERATE	Not significant
Complications (patients allocated)	229 per 1.000	254 per 1.000 (199 to 327)	RR 1.11 (0.87 to 1.43)	580 (3 RCTs)	⨁⨁⨁◯ MODERATE	Not significant
Reoperations (patients treated)	104 per 1.000	225 per 1.000 (150 to 336)	RR 2.17 (1.45 to 3.24)	630 (5 RCTs)	⨁⨁◯◯ LOW	Significant
Reoperations (patients allocated)	94 per 1.000	192 per 1.000 (128 to 288)	RR 2.05 (1.37 to 3.08)	730 (5 RCTs)	⨁⨁◯◯ LOW	Significant

*The risk in the intervention group (and its 95% confidence interval) is based on the assumed risk in the comparison group and the relative effect of the intervention (and its 95% CI).

CI: Confidence interval; RR: Risk ratio; MD: Mean difference; SMD: Standardized mean difference, ZCQ: Zurich Claudication Questionnaire; SS: symptom severity; PF: physical function; VAS: visual analogue scale

### Overall completeness and applicability of evidence

We included only RCTs in this review, which resulted in the inclusion of six trials (nine papers) with 930 participants. IPD implant was compared to conservative treatment (one trial) and surgery (minimally invasive surgery–one trial, decompression and arthrodesis–two trials, and decompression only–three trials).

Primary and secondary outcomes were not measured in all studies and were often published in incomplete form.

Subgroup analysis was not feasible.

Therefore, there was an important lack of data for our analysis and this limited the applicability of our findings.

### Quality of the evidence

The overall quality of the evidence was low, as the result of methodological flaws, such as absence of blinding, allocation concealment, and potential conflicts of interest. Not all papers reported data compatible with the CONSORT Statement standards[34].

Thus, quantitative results of this review should be interpreted with caution and require further confirmation.

### Potential biases in the review process

This review was conducted in conformity to previously published criteria (Prospero). Our search strategy was comprehensive, without language restriction. However, it is possible that we have missed some potentially eligible studies. Difficulty in contacting authors may have prevented us of including data in quantitative analysis.

A follow-up period of 2 years may be too short for a chronic degenerative disease such as DLSS. Although Musacchio et al[33] found similar results within 2 and 5 years of follow-up, the number of participants was too small to draw any conclusions. Kamala et al[35] reported a high symptoms recurrence rate after 24 months in patients submitted to IPD implant.

#### Agreements and disagreements with other studies or reviews

We found four previously published systematic reviews that strictly compared IPDs to decompressive surgery. However, only one addressed exclusively RCTs.

Wu et al.[18] included RCTs and non-randomized prospective studies, with a minimum of 30 patients, and follow-up of at least 12 months. They found no significant difference between IPDs and decompressive surgery in clinical outcomes (back pain, leg pain, ODI, RMQ). Quality of life was not assessed. The authors also found a significant higher rate of reoperation in the IPD group, with higher financial costs.

Hong et al.[20] considered RCTs and cohort trials, limited to the English language, and found similar results for the outcomes our study assessed, also with higher reoperation rates in patients treated with IPDs.

Phan et al.[17] included RCTs and prospective observational studies, limited to the English language. They found no superiority of IPDs compared to decompression and also found a higher reoperation rate in the IPD group.

Zhao et al.[19] considered only RCTs and included 4 trials. Their analysis indicated that IPDs had higher reoperation rates, higher back pain scores and worse cost-effectiveness compared with decompression.

A Cochrane Systematic Review conducted by Machado et al.[3] on all treatment options for DLSS reported that IPDs did not provide better outcomes than conventional decompression and were associated with higher reoperation rates.

## Conclusions

### Implications for research

All future trials should meet CONSORT criteria for the design and reporting of non-pharmacological studies[34]. Longer follow-up periods of more than 2 years should be pursued, and subgroup analysis based on age and symptom intensity could better clarify if there is a subpopulation of patients that may benefit from IPDs.

### Implications for practice

Evidence from one trial suggests that IPD provides better pain and functional outcomes that conservative treatment, but is associated to higher risk of complications.

Based on the evidence from five trials with high risk of bias, this review provided low-quality evidence that IPDs resulted in similar outcomes when compared to standard decompression surgery, and, in fact, patients submitted to IPD implants had significantly higher rates of reoperation, with lower cost-effectiveness. Until conclusive evidence becomes available, treatment options must be chosen very carefully on an individual patient basis, with full disclosure of unproven clinical benefits and presumably higher risk of reoperation.

## Supporting information

S1 Appendix PRISMA checklist. (DOC)
